# Immune-Related Sclerosing Cholangitis and Subsequent Pyogenic Liver Abscesses in Two Patients With Melanoma Treated by Triplet Therapy: A Case Report

**DOI:** 10.1097/CJI.0000000000000486

**Published:** 2023-09-19

**Authors:** Viola Schön, Daniel Stocker, Christoph Jüngst, Reinhard Dummer, Egle Ramelyte

**Affiliations:** * University of Zurich, Zurich, Switzerland; † Department of Dermatology, University Hospital Zurich, Zurich, Switzerland; ‡ Institute for Diagnostic and Interventional Radiology, University Hospital Zurich, Zurich, Switzerland; § Department of Gastroenterology and Hepatology, University Hospital Zurich, Zurich, Switzerland

**Keywords:** immune checkpoint inhibitors, programmed cell death ligand 1, targeted therapy, melanoma, immune-related toxicity

## Abstract

Immune checkpoint inhibitors have improved the treatment of many cancers. However, immune-related (IR) adverse events can limit their use. A rare but potentially severe IR adverse event is IR-cholangitis, which is mostly induced by anti-programmed cell death 1 (PD1) antibodies and is often corticosteroid-resistant. Consequently, immunosuppressive therapy is increased, which interferes with the antitumor response and bears the risk of infection. We report on 2 patients with BRAF V600E mutant melanoma, who presented with IR-sclerosing cholangitis under triplet therapy with atezolizumab [anti–programmed cell death ligand 1 (PD-L1) antibody], vemurafenib (BRAF inhibitor), and cobimetinib (MEK inhibitor). In both cases, the administration of corticosteroids initially resulted in a marginal improvement but was followed by a rebound of biliary enzymes and the subsequent emergence of pyogenic liver abscesses with bacteremia. Liver abscesses developed without preceding invasive procedures, which implies that a more restrictive approach to immunosuppressive therapy for IR-cholangitis should be considered. To our knowledge, we report the first 2 cases of IR-cholangitis and subsequent liver abscesses without prior invasive intervention, the first cases of IR-cholangitis induced by triplet therapy, and 2 of the few anti-PD-L1 induced cases contributing to the evidence that both anti-PD1 and anti-PD-L1 antibodies induce IR-cholangitis. Treatment strategies for IR-cholangitis need to be improved to prevent life-threatening infectious complications.

## BACKGROUND

Immune checkpoint inhibitors (ICIs) have revolutionized cancer therapy but exposed patients to a risk of immune-related adverse events (irAEs). Immune-related cholangitis (IR-cholangitis) is a rare but severe irAE, which typically presents in laboratory work-up with a more prominent elevation of biliary enzymes, that is, alkaline phosphatase (ALP) and gamma-glutamyl transferase (GGT), than transaminases.^1^ IR-cholangitis can be classified into 2 types based on the size of the bile ducts affected. The first type is IR-sclerosing cholangitis, which affects the large bile ducts and is characterized by biliary caliber irregularities with dilatations, strictures, and wall thickening visible in imaging. The second type is small-duct cholangitis, which lacks biliary caliber irregularities and is identified by pathologic features, such as portal inflammation and bile duct injury.^1^ For the management of IR-cholangitis, the European Society of Medical Oncology guidelines recommend corticosteroids (CS) and ursodeoxycholic acid (UDCA).^2^ However, biochemical response to CS is often incomplete, leading to dose increase or additional immunosuppressives and, hence, increased risk of infection.^1^

In this report, we present 2 challenging cases of IR-sclerosing cholangitis induced by triplet therapy with anti–programmed cell death ligand 1 (PD-L1) antibody, atezolizumab, and BRAF/MEK inhibitors, vemurafenib, and cobimetinib, which were complicated by pyogenic liver abscesses with bacteremia.

## CASE 1

A patient in her late 60s presented with a BRAF V600E mutated melanoma with lymph node and brain metastases. Her disease had previously progressed under ICIs and BRAF/MEK inhibitors (BRAFi/MEKi). In March 2021, we started triplet therapy (triplet) with atezolizumab, vemurafenib, and cobimetinib.

**FIGURE 1:**
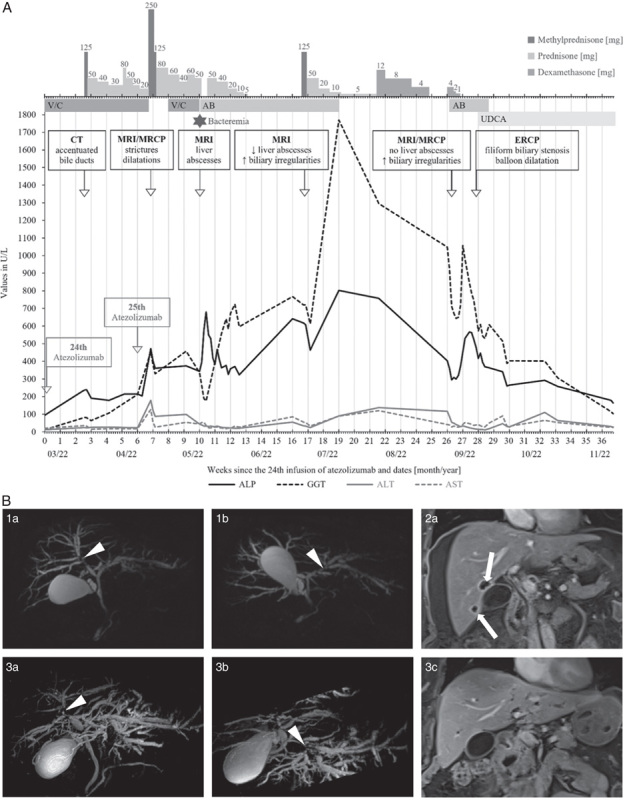
Clinical course and imaging of patient 1. A, The timeline starts when hepatobiliary enzymes were still normal. B, MRI/MRCP imaging of patient 1. (1a and 1b) Three-dimensional MRCP from initial MRI shows multiple strictures of the intrahepatic bile ducts (arrowheads) consistent with changes related to sclerosing cholangitis. (2a) Contrast-enhanced T1-weighted images 1 month later show multiple new peribiliary abscesses (arrows). (3a–c) On follow-up 3 months later, 3-dimensional MRCP images show persistent strictures of the left and right intrahepatic bile ducts (arrowheads in 3a and 3b) with increased dilatation. Contrast-enhanced T1 images show resolved peribiliary abscesses (3c). AB indicates antibiotics; ALP, alkaline phosphatase; ALT, alanine transaminase; AST, aspartate transaminase; ERCP, endoscopic retrograde cholangiopancreatography; GGT, gamma-glutamyl transferase; MRCP, magnetic resonance cholangiopancreatography; MRI, magnetic resonance imaging; UDCA, ursodeoxycholic acid; V/C, vemurafenib/cobimetinib.

In March 2022, after 12 months on triplet with 24 atezolizumab infusions, she presented with fever, fatigue, nausea, and right upper abdominal pain. Laboratory tests revealed elevated C-reactive protein and interleukin-6 and a grade 1 (G1) cholestatic liver injury according to the Common Terminology Criteria for Adverse Events v5.0. Her liver-related history was unremarkable. Computed tomography (CT) detected newly accentuated intrahepatic bile ducts without obstruction. Given inflammation without evidence of infection, a cytokine release syndrome was suspected. BRAFi/MEKi were withheld, and high-dose CS was initiated (Fig. 1A). Biliary enzymes persisted at G1 ALP and G3 GGT elevation; however, upon resolution of symptoms and inflammation within a week, she was dismissed. As CS was tapered to prednisone (PDN) 20 mg/d, she received the 25th atezolizumab infusion. Symptoms reappeared and laboratory tests revealed G3 cholestatic liver injury. Magnetic resonance imaging and cholangiopancreatography (MRI/MRCP) showed patchy enhancement of the liver parenchyma and intrahepatic bile duct dilatation with multisegmental strictures, suggestive of sclerosing cholangitis (Fig. 1B; 1a, b). Alternative causes of cholestatic liver disease were excluded (Supplemental Table 1, Supplemental Digital Content 1, http://links.lww.com/JIT/A786). Given the history and temporal association of the cholestatic liver injury with reexposure to atezolizumab, laboratory, and imaging findings, we diagnosed IR-sclerosing cholangitis. Despite withholding BRAFi/MEKi and administering high-dose CS, elevated hepatobiliary enzymes persisted (G2 ALP, G3 GGT, and G1 transaminases).

**FIGURE 2:**
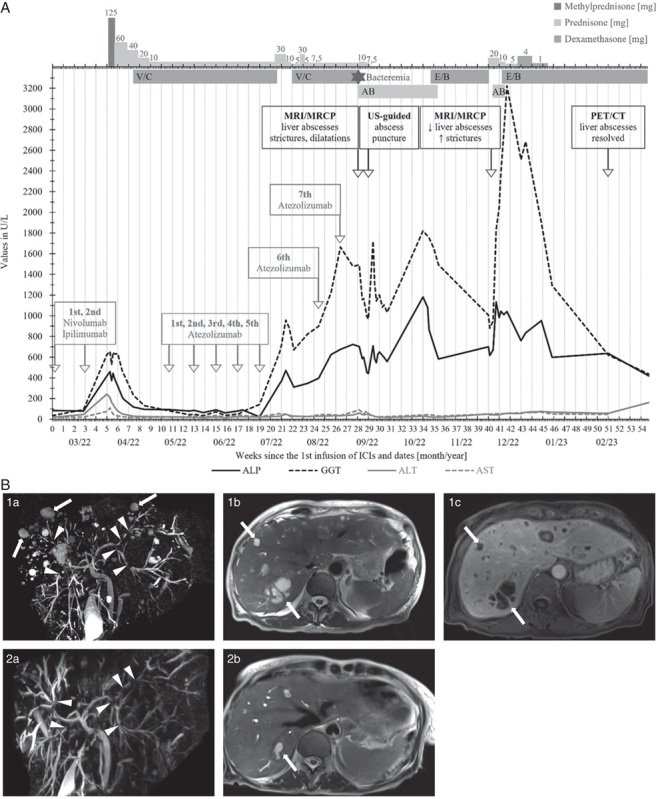
Clinical course and imaging of patient 2. A, The timeline starts at the initiation of immune checkpoint inhibitors. B, MRI/MRCP imaging of patient 2. (1a–c) Three-dimensional MRCP from the initial MRI shows multiple strictures of the intrahepatic bile ducts (arrowheads in 1a) and multiple round, hyperintense lesions on MRCP and T2-weighted images (arrows in 1a and 1b), which show discreet peripheral and septal contrast enhancement in T1-weighted images (arrows in 1c), consistent with hepatic abscesses. (2a and 2b) On follow-up 3 months later, MRCP shows persistent intrahepatic bile duct strictures (arrowheads in 2a) with increased dilatation, and T2-weighted images show decreased to completely resolved intrahepatic abscesses (arrow in 2b). AB indicates antibiotics; ALP, alkaline phosphatase; ALT, alanine transaminase; AST, aspartate transaminase; CT, computed tomography; E/B, encorafenib/binimetinib; GGT, gamma-glutamyl transferase; MRCP, magnetic resonance cholangiopancreatography; MRI, magnetic resonance imaging; PET, positron emission tomography; US, ultrasound; V/C, vemurafenib/cobimetinib.

In May 2022, during the seventh week under CS with current PDN 50 mg/d, she presented with fever, vomiting, diarrhea, right upper abdominal pain, G3 cholestasis, G1 transaminases, and inflammation (C-reactive protein, 320 mg/L; interleukin-6, 102.5 pg/mL; procalcitonin, 6.7 µg/L; leukocytosis). MRI showed multiple peribiliary liver abscesses (Fig. 1B; 2a), blood cultures detected *Morganella morganii* and concurrently severe *Clostridium difficile* colitis was diagnosed. She developed a septic shock and was transferred to the intensive care unit. BRAFi/MEKi were discontinued, antibiotics initiated for 9 weeks, and CS tapered over 2 weeks. Her clinical state ameliorated, the inflammation resolved, and she was dismissed after 3 weeks, however, with persistent G2 ALP and G3 GGT elevation.

Cholestatic liver injury exacerbated 3 weeks after cessation of CS in late June 2022 (G3 ALP, G4 GGT, and G2 transaminases). MRI displayed shrinkage of the liver abscesses but aggravated biliary strictures. Blood cultures were negative and inflammation moderate. She was rehospitalized, antibiotics were continued, and high-dose CS was readministered and tapered over 8 weeks. Being off triplet, brain metastases progressed, hence, stereotactic radiotherapy was conducted.

After 2 months on CS, cholestasis was persisting (G3 ALP, G4 GGT, and G1 transaminases). MRI/MRCP displayed resolved peribiliary abscesses but increased intrahepatic biliary strictures (Fig. 1B; 3a–c). Endoscopic retrograde cholangiopancreatography was conducted for balloon dilatation of the strictures, and UDCA 1 g/d was initiated. Consecutively, hepatobiliary enzymes descended slowly to G1 cholestasis and normal transaminases at the last follow-up in February 2023. Then, she had an ongoing complete extracranial response with intracranial oligoprogression. Hence, stereotactic radiotherapy was conducted again, and targeted therapy with encorafenib and binimetinib (BRAFi/MEKi) was initiated.

## CASE 2

A patient in her late 60s presented with a BRAF V600E mutated melanoma with cerebral, pulmonary, hepatic, adrenal, and bone metastases. She previously received 2 cycles of nivolumab and ipilimumab, which were discontinued in March 2022 due to G3 IR-hepatotoxicity with cholestatic pattern and tumor progression. Alternative causes of cholestatic liver disease were excluded (Supplemental Digital Content 1, http://links.lww.com/JIT/A786). Management with high-dose CS led to the normalization of hepatobiliary enzymes (Fig. 2A). Triplet therapy was initiated in April 2022, and partial response was achieved.

After the fifth atezolizumab infusion, still under PDN 10 mg/d for prevention of IR-hepatotoxicity, she presented with fever, fatigue, nausea, and inappetence. Laboratory tests revealed cholestatic liver injury with G2 ALP, G4 GGT, and G1 transaminases elevation. CT showed ongoing partial response without further pathologic findings. She was hospitalized for suspected IR-hepatitis. BRAFi/MEKi were withheld, and PDN increased to 30 mg/d and tapered to 7.5 mg/d over 2 weeks. However, elevated biliary enzymes persisted. Given the suspected diagnosis of IR-hepatitis and normalized transaminases, biliary enzyme elevation was deemed to be clinically insignificant, and triplet was resumed. However, the resumption of triplet led to worsening of the cholestatic liver injury with each atezolizumab infusion.

In August 2022, after 7 atezolizumab cycles and 23 weeks on CS, she was hospitalized with subicterus, fatigue, fever, and abdominal discomfort. Laboratory tests revealed cholestatic liver injury (G3 ALP, G4 GGT, and G1 transaminases) and inflammation (C-reactive protein 275 mg/L, leukocytosis 10.0 G/L). Liver MRI/MRCP showed regressive metastases, however, with multiple liver abscesses (Fig. 2B; 1a–c). Furthermore, intrahepatic biliary strictures and patchy enhancement of the liver parenchyma were seen, consistent with sclerosing cholangitis. Pus culture of the biggest liver abscess through ultrasound-guided puncture and blood cultures detected *Klebsiella oxytoca* and *Enterococcus faecium*. Given the development of cholestatic liver injury after ICI, rebound, and further progression with atezolizumab, and after excluding alternative causes (Supplemental Digital Content 1, http://links.lww.com/JIT/A786), IR-sclerosing cholangitis complicated with liver abscesses was diagnosed. Triplet and PDN were discontinued, and antibiotics were initiated for 9 weeks.

In October 2022, imaging showed tumor progression; hence, we initiated targeted therapy with encorafenib and binimetinib (BRAFi/MEKi). A month into therapy, she was hospitalized with fatigue, subfebrile temperature, cholestatic liver injury (G3 ALP, G4 GGT, and G1 transaminases), and moderate inflammation. MRI/MRCP showed nearly resolved liver abscesses but increased intrahepatic biliary dilatations and strictures (Fig. 2B; 2a, b). BRAFi/MEKi were withheld, antibiotics initiated, and CS tapered over 5 weeks administered. Although inflammation improved, cholestatic liver injury worsened (G3 ALP, G4 GGT, and G1 transaminases), and brain metastases progressed, for which she received stereotactic radiotherapy and dexamethasone. At the last follow-up in March 2023, liver abscesses had resolved in positron emission tomography/CT, and a complete extracranial metabolic response with partial intracranial response was achieved. The cholestatic liver injury had descended to G3 GGT, G2 ALP, and G1 transaminases elevation.

## DISCUSSION

To our knowledge, this is the first report of atezolizumab, vemurafenib, and cobimetinib-induced sclerosing IR-cholangitis, complicated by pyogenic liver abscesses and bacteremia without preceding invasive procedures. These cases raise the question of whether BRAFi/MEKi in this triplet may contribute to the pathogenesis of IR-sclerosing cholangitis and underline the need for better awareness and management strategies of rare irAEs.

IR-cholangitis has been reported in over 50 cases, mostly induced by anti–programmed cell death 1 (PD1) and far less by anti–PD-L1, with a median onset after 5 ICI cycles, ranging up to 27 cycles.^1,3,4^ The higher number of cases induced by anti-PD1 compared with anti–PD-L1, however, is most likely due to their earlier introduction to the market and consequently greater number of patients treated with anti-PD1.^5^ In our report in case 1, cholestatic liver injury emerged after 1 year on triplet therapy (24 atezolizumab cycles). In case 2, it emerged after 2 cycles of ipilimumab/nivolumab, however, was masked by concurrently elevated transaminases and reemerged after 3 months on triplet therapy (5 atezolizumab cycles). Cholestatic liver injury aggravated in both cases with further atezolizumab administrations and typical imaging findings of IR-sclerosing cholangitis were present. While cholangiopathy induced by protein kinase inhibitors was previously reported, to our knowledge, none were caused by selective BRAFi/MEKi.^5^ However, the known immune-modulating effects of BRAFi/MEKi may potentially aggravate autoimmune toxicity if given in combination with ICIs.^6^

In the present cases, biliary enzymes rebounded under CS, and both developed pyogenic liver abscesses with bacteremia. These complications were managed successfully with antibiotics. However, pyogenic liver abscesses can complicate biliary tract disease and are life-threatening with 6%–10% mortality rates.^7^ One fatal case of IR-cholangitis complicated with liver abscesses subsequent to immunosuppressives and endoscopic biliary stenting was reported.^8^ In the present cases, no preceding intervention was conducted, which implies that the bacterial cholangitis and liver abscesses occurred merely on the basis of IR-sclerosing cholangitis and subsequent CS therapy. Moreover, pyogenic cholangitis may further aggravate sclerosing cholangitis.^9^ This was observed in our cases, as biliary enzymes and strictures increased after the manifestation of the liver abscesses. Therefore, when biliary enzymes rebound during immunosuppressive therapy for IR-cholangitis, biliary infection needs to be excluded.^1,10^

As an attempt to reduce CS for the treatment of IR-cholangitis, UDCA is recommended, as it has shown positive effects on biliary enzymes in patients with IR-cholangitis and sclerosing cholangitis of other origin.^1,2^ In our first case, UDCA led to nearly normalized biliary enzymes upon balloon dilatation with endoscopic retrograde cholangiopancreatography despite discontinuation of CS. To clarify the effectiveness of UDCA and to reduce CS-related complications, long-term follow-up studies of patients with IR-cholangitis evaluating MRCP imaging and UDCA treatment would be of interest, as biochemical response to CS is often incomplete and protracted.^1^ Moreover, to prevent irreversible bile duct injury, it is crucial that hepatobiliary enzymes are assessed before every cycle of ICI therapy to detect cholestatic liver injury typical for IR-cholangitis early, and consequently, to withhold ICIs immediately.^2^

In conclusion, we report the first cases of IR sclerosing cholangitis complicated by pyogenic liver abscesses and bacteremia under CS without preceding invasive interventions. Awareness of IR-cholangitis, life-threatening infectious complications, and their management should be raised.

## CONFLICTS OF INTEREST/FINANCIAL DISCLOSURES

R.D. declares intermittent, project-focused consulting and/or advisory relationships with Novartis, Merck Sharp and Dhome (MSD), Bristol-Myers Squibb (BMS), Roche, Amgen, Takeda, Pierre Fabre, Sun Pharma, Sanofi, Catalym, Second Genome, Regeneron, Alligator, T3 Pharma, MaxiVAX SA, Pfizer, and touchIME outside the submitted work. E.R. has served as an advisor and/or received speaking fees and/or travel support from Amgen, BMS, Eli Lilly, MSD, Novartis, Pfizer, Pierre Fabre, Roche, Sanofi, and Philogen outside the submitted work. The remaining authors have declared that there are no financial conflicts of interest with regard to this work.

## Supplementary Material
