# Supplementary material for: Immune-Related Sclerosing Cholangitis and Subsequent Pyogenic Liver Abscesses in Two Patients With Melanoma Treated by Triplet Therapy: A Case Report
Source: J Immunother. 2023 Sep 19;46(9):346–50. doi: 10.1097/CJI.0000000000000486 (PMC10540752; doi:10.1097/CJI.0000000000000486)
Supplement: Supplementary file 1 [file cji-46-346-s001.pdf]

Supplemental Digital Content 1

Supplemental Table 1. Diagnostic work-up for exclusion of alternative causes of cholestatic liver disease. Further excluded were regular alcohol consumption, other recent medication changes, previous hepatic ischemia, and by imaging biliary obstruction such as cholelithiasis or tumor progression, steatohepatitis, and liver cirrhosis (normal fibroscan). n, normal range; - , negative; + , positive; n.a., not available; IgG and IgM, immunoglobuline G and M; ANA, antinuclear antibody; ANCA, anti-neutrophil cytoplasmatic antibody; MPO, myeloperoxidase; PR3, proteinase 3; AMA, anti-mitochondrial antibody; Anti-M2, anti-mitochondrial M2 antibody; ASMA, anti-smooth muscle antibody; HAV, hepatitis A virus; HBs, hepatitis B surface; HBc, hepatitis B core; HCV, hepatitis C virus; CMV, cytomegalovirus; EBV, Epstein-Barr virus; VZV, Varicella zoster virus.

Immunological and viral markers	Patient 1	Patient 2
**Autoimmune cholangitis and hepatitis**		
IgG serum	n	n
IgG subclasses 1-4 serum	n	n
IgM	n	n
ANA	-	-
ANA:cytoplasm	-	n.a.
ANCA	-	1:80 (N<1:40)
Anti-MPO	-	-
Anti-PR3	-	-
AMA	-	-
Anti-M2	-	-
ASMA (anti-smooth muscle antibody)	n.a.	-
Anti-actin	n.a.	-
**Viral Hepatitis**		
Anti-HAV IgG, IgM	n.a.	IgG +, IgM -
HBs antigen	-	-
Anti-HBs	-	-
Anti-HBc	-	+
HCV antigen	n.a.	-
Anti-HCV	-	-
CMV DNA	-	n.a.
Anti-CMV IgG, IgM	n.a.	IgG +, IgM -
EBV DNA	-	n.a.
Anti-EBV IgG, IgM	IgG +, IgM -	IgG +, IgM -
VZV DNA	-	n.a.
